# Intraclade Variability in Toxin Production and Cytotoxicity of Bacillus cereus Group Type Strains and Dairy-Associated Isolates

**DOI:** 10.1128/AEM.02479-17

**Published:** 2018-03-01

**Authors:** Rachel A. Miller, Jiahui Jian, Sarah M. Beno, Martin Wiedmann, Jasna Kovac

**Affiliations:** a Department of Food Science, Cornell University, Ithaca, New York, USA; University of Helsinki

**Keywords:** Bacillus cereus, cytotoxicity, foodborne pathogens

## Abstract

While some species in the Bacillus cereus group are well-characterized human pathogens (e.g., B. anthracis and B. cereus sensu stricto), the pathogenicity of other species (e.g., B. pseudomycoides) either has not been characterized or is presently not well understood. To provide an updated characterization of the pathogenic potential of species in the B. cereus group, we classified a set of 52 isolates, including 8 type strains and 44 isolates from dairy-associated sources, into 7 phylogenetic clades and characterized them for (i) the presence of toxin genes, (ii) phenotypic characteristics used for identification, and (iii) cytotoxicity to human epithelial cells. Overall, we found that B. cereus toxin genes are broadly distributed but are not consistently present within individual species and/or clades. After growth at 37°C, isolates within a clade did not typically show a consistent cytotoxicity phenotype, except for isolates in clade VI (B. weihenstephanensis/B. mycoides), where none of the isolates were cytotoxic, and isolates in clade I (B. pseudomycoides), which consistently displayed cytotoxic activity. Importantly, our study highlights that B. pseudomycoides is cytotoxic toward human cells. Our results indicate that the detection of toxin genes does not provide a reliable approach to predict the pathogenic potential of B. cereus group isolates, as the presence of toxin genes is not always consistent with cytotoxicity phenotype. Overall, our results suggest that isolates from multiple B. cereus group clades have the potential to cause foodborne illness, although cytotoxicity is not always consistently found among isolates within each clade.

**IMPORTANCE** Despite the importance of the Bacillus cereus group as a foodborne pathogen, characterizations of the pathogenic potential of all B. cereus group species were lacking. We show here that B. pseudomycoides (clade I), which has been considered a harmless environmental microorganism, produces toxins and exhibits a phenotype consistent with the production of pore-forming toxins. Furthermore, *B. mycoides/B. weihenstephanensis* isolates (clade VI) did not show cytotoxicity when grown at 37°C, despite carrying multiple toxin genes. Overall, we show that the current standard methods to characterize B. cereus group isolates and to detect the presence of toxin genes are not reliable indicators of species, phylogenetic clades, or an isolate's cytotoxic capacity, suggesting that novel methods are still needed for differentiating pathogenic from nonpathogenic species within the B. cereus group. Our results also contribute data that are necessary to facilitate risk assessments and a better understanding as to which B. cereus group species are likely to cause foodborne illness.

## INTRODUCTION

The Bacillus cereus group is a species complex of spore-forming Gram-positive bacteria, which are ubiquitously distributed throughout a number of environments (1). The current phylogeny organizes 9 species into 7 phylogenetic clades (2): (I) B. pseudomycoides, (II) B. wiedmannii, (III) B. anthracis and B. cereus (sensu lato), (IV) B. cereus sensu stricto and B. thuringiensis, (V) B. toyonensis, (VI) B. weihenstephanensis and B. mycoides, and (VII) B. cytotoxicus. Species within the B. cereus group have been previously associated with (i) outbreaks of foodborne illness (B. cereus sensu stricto [3] and B. cytotoxicus [2]), (ii) food spoilage (B. weihenstephanensis and B. mycoides [4]), (iii) anthrax in both humans and animals (B. anthracis [5]), and (iv) use as insecticides in agriculture (B. thuringiensis [6]) or (v) are considered nonpathogenic environmental microorganisms (B. pseudomycoides). The newest members of the B. cereus group include a putative probiotic species, B. toyonensis (7), and a psychrotolerant cytotoxic species, B. wiedmannii (8). While 9 additional species isolated from marine environments were recently proposed as novel members of the B. cereus group, these species were described after the completion of this study (9).

The U.S. Centers for Disease Control and Prevention (CDC) estimates that B. cereus group isolates are responsible for 63,400 (90% credible interval, 15,719 to 147,354) cases of foodborne illness in the United States each year (10). Compared to other pathogens which cause a more severe illness, the clinical presentation of B. cereus group foodborne illness is relatively mild and does not typically result in hospitalization (11), although serious complications have been documented (3, 12). Therefore, the true financial and public health burden attributed to B. cereus group species is likely underestimated (13, 14).

Phylogenies based on whole-genome sequence (WGS) data have identified several type strains, representing multiple species, which cluster into single phylogenetic clades with genome-wide DNA similarity values above the average nucleotide identity based on a BLAST (ANIb) species cutoff (15–17). Specifically, B. cereus sensu stricto clusters with B. thuringiensis into clade IV, and B. mycoides and B. weihenstephanensis cluster into clade VI. While this may suggest misclassification of type strains, it more likely represents issues associated with a phenotype-based taxonomy that is not associated with phylogeny. The incongruences between phylogenetic clades and taxonomic species assignments based on phenotypic traits represent a major challenge for the identification and classification of B. cereus group isolates, and specifically, the development of reliable identification methods for identifying strains likely to cause illness in humans.

While foodborne illnesses caused by B. cereus group isolates have been linked to a number of foods, particularly rice and meat dishes (18), spores and vegetative cells of B. cereus group isolates are also regularly isolated from dairy foods (e.g., fluid milk [19, 20]) and dairy-related environments (e.g., animal bedding [21, 22] and feed [23]), which can be sources of spore contamination of raw milk (24–26). As B. cereus group spores present in raw milk may not be inactivated by high-temperature short-time (HTST) pasteurization (72°C for 15 s), they can germinate and potentially grow to high levels in pasteurized fluid milk and refrigerated dairy products (25, 27–30). This is of particular importance, as multiple B. cereus group species (e.g., B. mycoides, B. weihenstephanensis, and B. wiedmannii) have been shown to grow at temperatures as low as 6 to 7°C (8, 31), although characterizations of their pathogenic potential remain discrepant (1, 16, 32, 33). Consequently, at least some B. cereus group species and strains represent potential safety and food spoilage hazards in dairy products, such as fluid milk (20).

The standard method for identifying B. cereus group isolates in food products in the United States involves plating the food sample on media selective and/or differential for B. cereus group species (34), followed by biochemical analyses to differentiate between species in the group (35, 36), although characterizations documenting the strain-to-strain variability are lacking. Taxonomic challenges have been overcome to some extent by using molecular approaches (e.g., *panC* or *rpoB* sequencing, 7-gene multilocus sequence typing [MLST], and WGS [2, 15, 16, 33]), which can reliably classify B. cereus group isolates into 7 phylogenetic clades. However, rapid differentiation between pathogenic and nonpathogenic strains remains an important challenge, particularly since the presence of single toxin genes or a set of toxin genes does not necessarily indicate that a specific strain is likely to cause human disease. For example, diarrheagenic strains are difficult to identify, as B. cereus group isolates contain multiple diarrheal toxin-encoding genes, including those for hemolysin BL (Hbl; encoded by *hblCDA* [37]), the nonhemolytic enterotoxin (Nhe; encoded by *nheABC* [38, 39]), and cytotoxin K encoded by either *cytK-1* (gene variant specific to B. cytotoxicus [40, 41]) or *cytK-2* (42). It has also been shown that not all toxins are essential to cause illness, as B. cytotoxicus, a B. cereus group species associated with several foodborne outbreaks (40, 41), lacks genes encoding Hbl and Nhe but is still pathogenic in humans (15, 40, 43). Importantly, different toxin genes have been identified in isolates representing several species and phylogenetic clades (15, 16, 44), indicating that strains from a number of phylogenetic clades may have the genetic capacity to cause foodborne disease. However, the associations between virulence genes and factors influencing their expression, cytotoxicity, and virulence have not been systematically explored (45).

The inability to reliably differentiate pathogenic from nonpathogenic B. cereus group isolates represents a major challenge for both regulatory agencies and food processors, including the dairy industry, as B. cereus group species are commonly isolated from and have been shown to grow in fluid milk and dairy products (20, 46). Thus, we (i) applied genetic and phenotypic characterization methods to a phylogenetically diverse set of 44 B. cereus group dairy-associated isolates and 8 B. cereus group type strains, (ii) assessed their cytotoxic potential at 37°C (human body temperature), and (iii) evaluated associations between phenotypic and/or genetic markers associated with cytotoxicity at 37°C to allow for a rapid prediction of the virulence potential of isolates from specific clades. Our data presented here demonstrate tremendous intraclade variability in phenotypic traits used for differentiation as well as the detection of toxin genes and their protein products, and they highlight that multiple clades contain isolates which are cytotoxic toward human cells.

## RESULTS

### Dairy foods and environments harbor phylogenetically diverse B. cereus group isolates.

**FIG 1:**
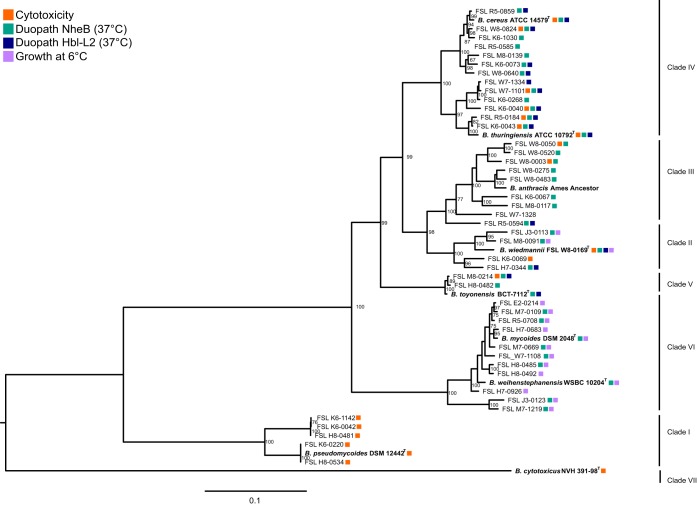
Phylogenetic relatedness of 44 dairy-associated B. cereus group isolates and 9 type strains. The maximum likelihood tree, rooted by midpoint, was constructed in RAxML based on core genome SNPs using a generalized time reversible model and 1,000 bootstrap repetitions. Bootstrap values of ≥60 are displayed on the branches. The bar represents substitutions per site. Type strains representative of the different B. cereus group species are indicated by bold text and are included in the phylogenetic tree as a reference. Dark-blue boxes represent isolates which were Hbl-L2 positive (Duopath Hbl-L2), green boxes represent isolates which were NheB positive (Duopath NheB), orange represents isolates which were cytotoxic in a HeLa cell model, and purple represents isolates able to grow at 6°C. Phenotypic results were not obtained for B. anthracis.

To capture the genetic and phenotypic diversity of strains within the B. cereus group, we compiled a collection of 44 B. cereus group isolates (see Table S1 in the supplemental material) obtained from fluid milk and dairy-associated environments throughout the northeastern United States between 2005 and 2016 (46–50). Characterization of isolates representing the diversity of the B. cereus group population from these sources is particularly relevant, as B. cereus group isolates have been previously associated with foodborne disease and the spoilage of dairy products (1, 4, 20). An additional 9 B. cereus group species type strains (the WGS for the B. anthracis Ames ancestor was included as the representative genome for B. anthracis) were included in phylogenetic analyses to serve as a reference. Together, these 53 isolates clustered in 7 phylogenetic clades (Fig. 1) based on a core genome single-nucleotide polymorphism (SNP) maximum likelihood phylogenetic tree; these clades were named to be consistent with previously defined phylogenetic groups (2, 15–17). Four clades included a single type strain and therefore allowed phylogeny-based species classification of isolates. These are (i) clade I with B. pseudomycoides DSM 12442^T^ and 5 dairy isolates, (ii) clade II with B. wiedmannii FSL W8-0169^T^ and 4 dairy isolates, (iii) clade V with B. toyonensis BCT-7112^T^ and 2 dairy isolates, and (iv) clade VII with just B. cytotoxicus NVH 391-98^T^. Clade IV includes both B. cereus sensu stricto ATCC 14579^T^ and B. thuringiensis ATCC 10792^T^ as well as 13 dairy isolates. Clade VI includes both B. mycoides DSM 2048^T^ and B. weihenstephanensis WSBC 10204^T^ as well as 11 dairy isolates. Clade III includes the B. anthracis Ames ancestor and 9 dairy isolates. No genes encoding B. anthracis plasmid-encoded virulence factors (e.g., *lef*, *cya*, and capsular genes *capABCDE*) were identified in any of the 9 dairy isolates in this clade; it has been shown previously that isolates in clade III (B. anthracis/B. cereus sensu lato) do not always carry the anthrax-associated plasmid-encoded virulence factors and hence cannot always be identified as B. anthracis (16, 51, 52).

### The majority of traditional phenotypic traits used for the characterization of B. cereus group isolates are shared by isolates from multiple phylogenetic clades and are not recommended for clade assignment.

**FIG 2:**
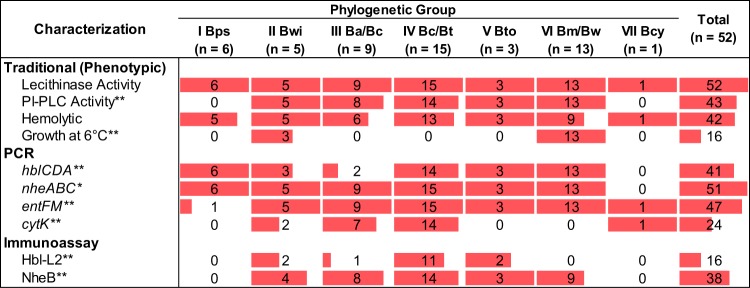
Variability in phenotypic and molecular characteristics among isolates in different B. cereus clades. Shown is a summary of the number of isolates positive for a given phenotypic or molecular characteristic by clade. The proportion of each cell that is shaded in red corresponds to the proportion of the isolates within a given clade which were positive for the phenotype (i.e., a fully shaded cell corresponds to all isolates being positive for that phenotype). Isolates were classified as PCR positive for gene operons if at least one gene in a given operon was detected by PCR. Asterisks correspond to *P* values obtained from Fisher's exact tests to assess differences in proportions of isolates positive for a given characteristic in a specific phylogenetic clade compared to isolates positive for the characteristic among all of the other clades combined. **, *P* < 0.001; *, *P* < 0.05. Bps, B. pseudomycoides; Bwi, B. wiedmannii; Ba/Bc, *B. anthracis/B. cereus sensu lato*; Bc/Bt, *B. cereus sensu stricto/B. thuringiensis*; Bto, B. toyonensis; Bm/Bw, *B. mycoides/B. weihenstephanensis*; Bcy, B. cytotoxicus.

While current standard methods for the differentiation of B. cereus group species rely on phenotypic characterization data (34, 35), systematic phenotypic characterization studies on type strains and environmental isolates are still needed to describe the diversity of phenotypes represented by isolates in each clade. We thus performed phenotypic characterizations of the 44 dairy-associated isolates and 8 type strains (B. anthracis was not characterized), which showed that all B. cereus group isolates characterized here have lecithinase activity (Fig. 2), and the majority of isolates (42 out of 52) have hemolytic activity. Phosphoinositide phospholipase C (PI-PLC) activity was detected for 43 out of 52 isolates, and a subset of isolates (16 out of 52) were able to grow at 6°C. Fisher's exact tests were used to assess whether proportions of phenotype-positive isolates within a given clade were significantly different from those in other clades. Both PI-PLC activity and growth at 6°C were significantly associated with clade classification (*P* < 0.001 for both; see Table S2). Specifically, the absence of PI-PLC activity was significantly associated with clade I (B. pseudomycoides; 0 out of 6 isolates in this clade were positive for PI-PLC activity). The ability to grow at 6°C was exclusive to isolates in clades II (B. wiedmannii; 3 out of 5 isolates) and VI (B. mycoides/B. weihenstephanensis; 13 out of 13 isolates).

### The majority of toxin genes are widely distributed across multiple clades.

All isolates were tested via PCR for the presence of key toxin genes to (i) probe the distribution of these genes among clades and (ii) determine the relationships between phenotypic (i.e., detection of toxin proteins and cytotoxicity in HeLa cells) and genetic characteristics (i.e., presence of toxin genes). Overall, 51 out of 52 isolates tested contained at least one of the Nhe genes (i.e., *nheA*, *nheB*, or *nheC*); only the B. cytotoxicus type strain (clade VII) was negative for all 3 Nhe genes, which agrees with what has been reported previously (15). While *nheC* was detected by PCR for all 51 isolates in clades I to VI (only the type strain in clade VII was negative), *nheA* and *nheB* were detected less frequently (41 out of 52 and 45 out of 52 isolates, respectively [Table S3]). *entFM* was also broadly distributed (47 out of 52 isolates were positive). Interestingly, all 5 *entFM* PCR-negative isolates were in clade I (B. pseudomycoides); all dairy-associated isolates in this clade were PCR negative for *entFM*, and DSM 12442^T^ was the only *entFM*-positive isolate in this clade. Genes encoding hemolysin BL (*hblCDA*) were detected in 41 out of 52 isolates; isolates were classified as positive if at least one Hbl gene was detected by PCR (Fig. 2). *hblD* was the most commonly detected Hbl toxin gene (41 out of 52 isolates), while *hblA* and *hblC* were both detected in 39 out of 52 isolates screened (Table S3); 37 out of 52 isolates were positive for all Hbl genes. *cytK* (both variants 1 and 2 were detected by the primers used here) was detected in 24 out of 52 isolates. All isolates positive for *cytK* represented clades II, III, IV, and VII (Fig. 2 and Table S3).

For all toxin genes (*cytK* and *entFM*; for the Nhe and Hbl toxin gene operons, only one gene had to be detected for the isolate to be classified as positive), the proportions of isolates which were PCR positive for toxin genes were significantly different (all *P* < 0.05; Table S2) among clades. *Post hoc* analyses further identified specific clades that showed significant associations (*P* < 0.05) with the presence/absence of toxin genes (Table S2). Specifically, clade I (B. pseudomycoides) had lower proportions of *entFM*- and *cytK*-positive isolates, clade III (*B. anthracis/B. cereus sensu lato*) had a lower proportion of *hblCDA*-positive isolates, clade IV (*B. cereus sensu stricto/B. thuringiensis*) had a higher proportion of *cytK*-positive isolates, clade VI (B. mycoides/B. weihenstephanensis) had a higher proportion of *hblCDA*-positive isolates but a lower proportion of *cytK*-positive isolates, and clade VII (B. cytotoxicus) had a lower proportion of *nheABC*-positive isolates than all other clades combined.

To confirm that the lack of detection of a single gene within an operon (e.g., detection of *hblD* but not *hblC*) was not due to PCR-related issues, we also used BLAST detection of virulence genes in genomic sequences using BTyper, an open-source B. cereus group subtyping tool for the detection of virulence genes using WGS data (44). In total, there were 9 instances where BTyper detected the presence of a toxin gene even when the isolate was PCR negative (Table S4). Specifically, for Hbl genes, one additional isolate from clade VI (*B. mycoides/B. weihenstephanensis*) was *hblA* positive, and one additional isolate from IV (*B. cereus sensu stricto/B. thuringiensis*) was *hblC* positive. For *nhe* genes, 4 additional clade VI isolates were identified as *nheA* positive, and *nheB* was detected in 3 additional clade I (B. pseudomycoides) isolates. Overall, 80 out of 89 PCR-negative results (89.9%) were confirmed by WGS data analysis.

### NheB was detected in the majority of isolates containing *nheB*, with the exception of clade I isolates.

While previous studies had asserted the broad distribution of toxin-encoding genes among isolates from all clades (15, 53), it was unknown whether the production of Nhe and Hbl occurred in all clades. To correlate the production of Nhe and Hbl toxins with (i) phylogenetic clade and (ii) the results of the PCR detection of toxin genes, we used a commercially available lateral flow immunoassay (Duopath cereus enterotoxins) to detect Hbl lytic component L2 (Hbl-L2; encoded by *hblC*) and Nhe subunit NheB (encoded by *nheB*) production at 37°C.

Overall, 16 out of 52 isolates tested positive for Hbl-L2 with the lateral flow immunoassay; among the 39 *hblC* PCR-positive isolates, 15 isolates also tested positive for Hbl-L2 (Fig. 2). Hbl-L2 was not detected for any of the 6 and 13 isolates classified into clades I (B. pseudomycoides) and VI (*B. mycoides/B. weihenstephanensis*), respectively, despite all of these isolates (with the exception of one clade I isolate) being PCR positive for *hblC*. Clade VII (B. cytotoxicus) was also negative for Hbl-L2, but isolates in this clade do not encode Hbl (15, 40). NheB was detected for 38 out of 52 isolates; all 38 NheB-positive isolates were also PCR positive for *nheB*. NheB was detected broadly, with at least one NheB-positive isolate in all clades except for clades I and VII.

Because multiple isolates contained *hblC* (encoding Hbl-L2) and/or *nheB* (encoding NheB) but the corresponding protein products were not detected, we constructed phylogenetic trees based on *hblC* and *nheB* sequences extracted from the WGS data to assess if sequence diversification for these genes could account for the protein not being detected by the immunoassay.

**FIG 3:**
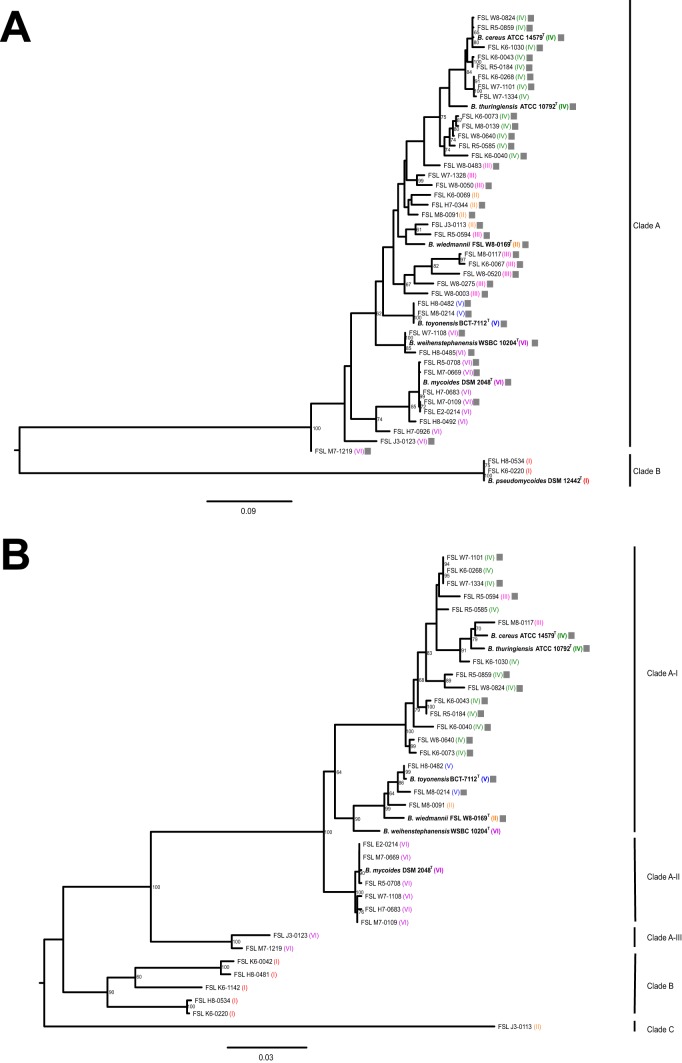
Lack of detection of NheB and Hbl-L2 toxin subunits among isolates with phylogenetically diverse sequences of *nheB* and *hblC*. Maximum likelihood trees were constructed for *nheB* (A) and *hblC* (B) based on DNA sequences extracted from WGS data for all isolates included in the phenotypic characterizations. Sequences were extracted from WGS data using a threshold of at least 40% nucleotide identity and 90% query coverage. Trees were constructed using the generalized time-reversible substitution model (GTR) and 1,000 bootstrap repetitions in RAxML and were rooted by midpoint. Bootstrap values of ≥60 are displayed on the branches. The bar in each panel represents substitutions per site. Gray boxes represent isolates that were positive for NheB (A) or Hbl-L2 (B) using the Duopath kit.

*nheB* sequences clustered into 2 clades; *nheB* clade A contained isolates from all phylogenetic clades, with the exception of 3 clade I (B. pseudomycoides) isolates (Fig. 3A), which clustered into *nheB* clade B. *hblC* phylogenetic trees clustered isolates into 3 main clades named clades A to C (Fig. 3B). Within clade A, 3 subclades (A-I, A-II, and A-III) were assigned. *hblC* clade A contained isolates from all B. cereus group phylogenetic clades except for clade I (*n* = 5 isolates; B. pseudomycoides) and 2 and 1 isolates from clades VI (*B. mycoides/B. weihenstephanensis*) and II (B. wiedmannii), respectively. All 15 Hbl-L2-positive isolates grouped into subclade A-I. Isolate FSL H7-0344 (clade II) was PCR negative, and an *hblC* sequence was not detected in the genome sequence for this isolate despite being Hbl-L2 positive using the immunoassay.

### All clades, with the exception of clade VI, have isolates that are cytotoxic at 37°C.

**FIG 4:**
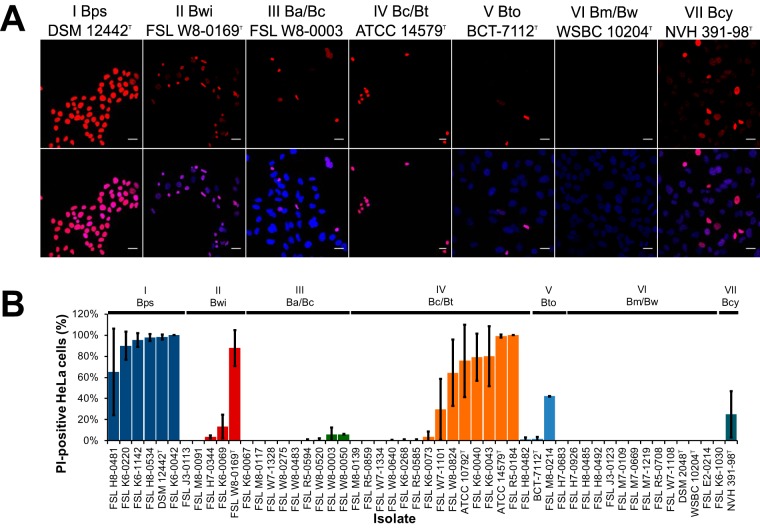
Isolates from multiple clades have cytotoxic activity *in vitro*. Fifty-two isolates were tested for the presence of biologically active pore-forming toxins present in culture supernatants. HeLa cells grown on coverslips were incubated with 5% (vol/vol) culture supernatants of each bacterial isolate and 5 μg of PI for 30 min. Cells were fixed with 4% formaldehyde and then nucleic acids were stained with 1 μg/ml DAPI as a control. All nuclei stained with DAPI (blue), while only cells with compromised membranes had nuclei that stained with PI (red). (A) Representative images for cytotoxicity assays performed for type strains (clade IV strain B. cereus ATCC 14579^T^ is shown and B. thuringiensis ATCC 10792^T^ is not; clade VI strain B. weihenstephanensis strain WBSC 10204^T^ is shown, B. mycoides strain DSM 2048^T^ is not) and FSL W8-0003 (representing clade III isolates [B. anthracis/B. cereus sensu lato]). DAPI and PI images represent the proportion of HeLa cells that were damaged due to cytotoxic activity of the respective bacterial isolate. Scale bar represents 20 μm. (B) Bar chart showing quantification of the proportions of HeLa cells having compromised cell membranes (PI positive) following coincubation with each of the 52 bacterial supernatants. Results represent the mean of 2 independent experiments. Error bars represent standard deviations. Bps, B. pseudomycoides; Bwi, B. wiedmannii; Ba/Bc, B. anthracis/B. cereus sensu lato; Bc/Bt, B. cereus sensu stricto/B. thuringiensis; Bto, B. toyonensis; Bm/Bw, B. mycoides/B. weihenstephanensis; Bcy, B. cytotoxicus.

To assess the pathogenic potential of the B. cereus group isolates, we used a modified propidium iodide (PI) exclusion assay to determine if coincubation with culture supernatants resulted in decreased membrane integrity in human epithelial (HeLa) cells due to exposure to pore-forming toxins (42). Overall, 19 out of 52 isolates were classified as cytotoxic, as indicated by there being ≥5% of PI-positive HeLa cells following coincubation with bacterial supernatants (Fig. 4). The proportion of isolates classified as cytotoxic differed significantly between clades (*P* < 0.001). None of the 13 clade VI (*B. mycoides/B. weihenstephanensis*) isolates were cytotoxic (significantly lower proportion of cytotoxic isolates than other clades; *P* = 0.002), while all other clades had at least one isolate with cytotoxic activity (Fig. 4). Interestingly, clade I (B. pseudomycoides) isolates had a significantly higher proportion of cytotoxic isolates (*P* = 0.001); all 6 isolates were highly cytotoxic, with a mean of 91% PI-positive HeLa cells following coincubation with supernatants from these isolates (Fig. 4B).

### No associations were found between the presence of toxin genes, protein expression, and isolate cytotoxicity.

Based on the previous establishment of Hbl, Nhe, and CytK toxins as pore-forming toxins (38, 39, 43, 54), the effects of which are detectable by the cytotoxicity assay described here, we used Fisher's exact tests to assess associations between isolate cytotoxicity and the detection of individual toxin genes and proteins. The detection of toxin proteins Hbl-L2 or NheB was not significantly associated with classification of an isolate as cytotoxic (*P* > 0.05). Similarly, PCR detection of pore-forming toxins *nheABC*, *hblCDA*, and *cytK-2* was not significantly associated with cytotoxicity (all comparisons *P* > 0.05; all *P* values are listed in Table S2).

## DISCUSSION

Using a combination of phenotypic, genetic, and cellular cytotoxicity data, we characterized a diverse collection of both environmental and type strains to provide updated information about toxin gene distribution and the pathogenic potential of B. cereus group isolates, with a specific focus on isolates from dairy-associated sources, as this is a pathogen that has been previously isolated from the dairy environment (19, 20, 28). Importantly, our study also included isolates that represent a new species (clade II, B. wiedmannii), as well as clades that had not been included in previous studies (clade I, B. pseudomycoides). Our results demonstrate that even though toxin genes and associated phenotypic characteristics can be found across isolates from essentially all B. cereus group clades, many of these characteristics are not consistently found within a given clade. Characterization of the pathogenic potential of isolates is further complicated by the fact that there does not seem to be a clear correlation between genetic markers (i.e., toxin gene presence/absence) and relevant phenotypic characteristics, such as tissue culture-based cytotoxicity assays. Importantly, our data show that the majority of clades contain isolates with cytotoxic activity. More specifically, we show that (i) isolates from clade VI (*B. mycoides/B. weihenstephanensis*) are not cytotoxic when cultured at 37°C, suggesting that isolates in this clade may be less likely to cause foodborne illness, and that (ii) clade I (B. pseudomycoides) isolates should be considered potential foodborne pathogens.

### Broad distribution of phenotypic traits among clades and phenotypic heterogeneity within clades suggests that phenotypic characterization of B. cereus group isolates has limited value for differentiation and species identification.

Traditionally, the identification and differentiation of B. cereus group species relied on classical phenotypic methods, such as hemolysis (with species B. anthracis being nonhemolytic [35]), lecithinase activity (present in all B. cereus group species [34]), and growth at 7°C but not at 43°C (considered diagnostic for B. weihenstephanensis [55]). Here, we present updated phenotypic characterizations of isolates from all 7 phylogenetic clades, including species type strains and a diverse collection of isolates from dairy-associated sources. In combination with previous studies characterizing type strains (7, 8, 40, 56–58), our results show a broad distribution of some phenotypic characteristics across multiple if not all clades (e.g., lecithinase), along with heterogeneity of a number of phenotypes within a given clade (e.g., hemolysis). This highlights the limited value of approaches based solely on phenotypic characteristics for identification and differentiation of B. cereus group clades or species. Our results do, however, suggest that lecithinase activity is a trait that all B. cereus group isolates share, including isolates from additional phylogenetic clades (e.g., clades I [B. pseudomycoides], II [B. wiedmannii], V [B. toyonensis], and VII [B. cytotoxicus]) that were not included previously (34), confirming that isolates from all 7 B. cereus group clades have a characteristic colony morphology on Bacara agar. On the other hand, PI-PLC activity showed a clade-specific pattern and was absent in all clade I (B. pseudomycoides) and clade VII (B. cytotoxicus) isolates tested here. It is important to emphasize that we also showed that the ability to grow at 6°C, which has been previously established as characteristic for clade VI (*B. mycoides/B. weihenstephanensis*) (8, 59), is also shared by some isolates in clade II.

### There are no clear associations between detection of toxin genes or toxin gene products and cytotoxicity.

Hypothesis-driven testing for PCR detection of toxin genes (i.e., *hblCDA*, *nheABC*, and *cytK*) and immunoassay detection of NheB or Hbl-L2 toxin subunits did not support any significant relationships (*P* > 0.05) with cytotoxicity. This is consistent with previous studies that have suggested that multiple B. cereus group toxins are important for cytotoxicity, based on various cellular assays (40, 54, 60, 61). It is thus possible that different combinations of toxins are responsible for a cytotoxicity phenotype (43). Nevertheless, the cytotoxicity data generated here do suggest that B. cereus group isolates in addition to clades IV (*B. cereus sensu stricto/B. thuringiensis*) and VII (B. cytotoxicus) may need to be considered in risk assessments that address the foodborne illness associated with members of the B. cereus group. Future studies that examine the regulation of Hbl, Nhe, and cytotoxin K toxins among isolates belonging to different clades will be key to understanding the contributions of these toxins to *in vitro* cytotoxicity and *in vivo* virulence. Overall, the observed heterogeneity of cytotoxicity among isolates in multiple B. cereus clades without a clear association between genetic and phenotypic data suggests the importance of regulatory mechanisms, such as activity of the virulence gene regulator PlcR (62), or other regulatory elements which have previously been associated with virulence factor expression (63, 64). Alternatively, the observed cytotoxicity characterized here could result from the production of unidentified toxins in isolates where discrepant results between the detection of genes and proteins and cytotoxicity were observed.

### Cytotoxicity data support the idea that clade I isolates produce pore-forming toxins and are potentially pathogenic, despite being negative for the detection of Hbl and Nhe using immunoassay kits developed for B. cereus sensu stricto.

Our data show that isolates from clade I (B. pseudomycoides) have high cytotoxic activity, as all 6 strains tested, including the type strain, resulted in a mean of 91% PI-positive HeLa cells following incubation with 5% (vol/vol) supernatant from B. pseudomycoides cultures. Our finding that clade I isolates are potentially pathogenic was surprising, given that clade I isolates have not been characterized in previous studies examining the cytotoxicity of B. cereus group isolates (32, 45). Importantly, we show that the cytotoxicity was observed for all clade I isolates obtained from dairy-associated environments, as well as for the B. pseudomycoides type strain. Interestingly, the toxin kit used here failed to detect NheB and Hbl-L2 subunits in supernatants from clade I isolates, despite these isolates containing *nheB* and *hblC*. Possible explanations for these findings include (i) these isolates produce Hbl and Nhe toxins which are not detected due to a lack of antibody recognition resulting from the protein subunits having an altered conformation, which has been shown previously for this immunoassay (65); or (ii) toxins other than Hbl and/or Nhe are responsible for the observed cytotoxicity, even though these isolates do not carry *cytK*, a likely candidate as an alternative cytotoxin (15). Our data suggest that further characterization of clade I isolates will be essential for understanding the true pathogenicity of clade I isolates.

### Clade VI represents the only clade with isolates that are not cytotoxic at 37°C.

Importantly, we show that all clades except clade VI (*B. mycoides/B. weihenstephanensis*) include at least some isolates that produce pore-forming toxins at 37°C, suggesting that isolates other than well-established foodborne pathogens that are represented by clades IV (*B. cereus sensu stricto/B. thuringiensis*) and VII (B. cytotoxicus) may have the ability to cause foodborne illness. This is consistent with a recent study that reported that strains from multiple B. cereus group clades have been previously implicated as the causative agent in foodborne outbreaks (66). Among the isolates characterized here, all clade VI isolates were capable of growing at 6°C, but none of these isolates were cytotoxic in tissue culture when grown at 37°C. Furthermore, despite the fact that all 13 clade VI isolates tested in this study contained *hblCDA*, none of them produced detectable Hbl-L2 at 37°C. Although 9 out of 13 isolates in clade VI produced detectable NheB, coincubation of HeLa cells with supernatants from isolates in this clade did not result in PI uptake indicative of exposure to pore-forming toxins. This suggests that clade VI isolates do not produce sufficient levels of pore-forming toxins at 37°C to be detected by the sensitive HeLa cell cytotoxicity assay described here. Interestingly, by applying bacterial supernatants to Caco-2 cell monolayers, Guinebretière et al. (32) found that some isolates from clade VI had low-level cytotoxic activity, although in that study, the isolates were cultured at 32°C instead of 37°C. The cytotoxicity of B. weihenstephanensis isolates grown at lower temperatures was also reported by Réjasse et al. (33). Furthermore, using a different cell model, Christiannson et al. (67) reported that supernatants from B. weihenstephanensis isolates (with clade not specified) cultured in milk at 8°C had cytotoxic activity (toxicity at a titer of 1:128) in human embryonic lung cells (67). Although our results suggest that clade VI isolates pose less of a public health hazard, as they are not cytotoxic in HeLa cells when grown at 37°C, further characterization of clade VI isolates, including studies on virulence gene expression under different conditions and at different temperatures, will be valuable to further assess the potential food safety risks associated with clade VI isolates. It is important, however, to emphasize that there is some information suggesting that clade VI isolates have been linked with foodborne outbreaks in the past (66), suggesting that some level of food safety risk is associated with these isolates. Furthermore, the ability of clade VI isolates to grow at 6°C, combined with their documented proteolytic activity (58) and causation of sweet curdling in milk (68), supports that these B. cereus group strains are important as food spoilage organisms.

### Conclusion.

The integration of phenotypic, genomic, and cytotoxicity data demonstrates that all B. cereus group clades, with the exception of clade VI (representing *B. mycoides/B. weihenstephanensis* isolates), include isolates that produce cytotoxic factors when grown at 37°C and therefore are potentially pathogenic. No single phenotypic characteristic is unique to any given B. cereus group clade, and most phenotypic traits are varied among isolates belonging to the same phylogenetic clade. This highlights that methods relying solely on phenotypic detection of B. cereus are not recommended for differentiation within the B. cereus group. While further testing of the regulatory mechanisms of toxin gene expression will be beneficial for developing predictive tools for an accurate assessment of B. cereus group isolates' pathogenic potential, the data provided here suggest that isolates from the majority of clades and species in the B. cereus group (including B. pseudomycoides) are potentially capable of causing foodborne illness.

## MATERIALS AND METHODS

### Isolates characterized in this study.

B. cereus group isolates (*n* = 52; Table S1) for this study included isolates which were selected from the Food Microbe Tracker (FMT) strain collection (69) and type strains of B. cereus group species. Isolates from the FMT were selected from B. cereus group isolates that had been isolated from dairy foods and dairy-associated environmental sources between 2005 and 2016 and that had also been previously characterized by sequencing of a 632-nucleotide segment of *rpoB*, which encodes the β-subunit of the RNA polymerase (20). The isolates characterized here were obtained from raw milk (20, 46, 47, 49), HTST pasteurized milk (50), the dairy farm environment (49), dairy foods collected at intermediate processing steps (48), and a dairy powder sample (48). Dairy-associated isolates were selected to include at least one representative for each of the 42 unique *rpoB* allelic types (ATs) found among the approximately 500 B. cereus group dairy isolates (see Table S1 for isolate details). Additional metadata, including MLST data collected here, for these isolates are available through the FMT online database (Cornell University, Ithaca, NY) (69). All isolates were preserved in brain heart infusion (BHI) broth (BD, Franklin Lakes, NJ) supplemented with 15% (vol/vol) glycerol and maintained at −80°C.

### Culturing conditions and colony morphology on selective differential media.

Isolates were substreaked from frozen glycerol stocks onto BHI agar and incubated for 20 to 24 h at 32°C. Single colonies were subsequently inoculated into BHI broth, followed by incubation at 32°C for 14 to 18 h. These cultures were diluted in phosphate-buffered saline (PBS) prior to spread plating on (i) BHI agar to assess general colony morphology, (ii) Bacara agar (AES Chemunex, Cranbury, NJ) to detect lecithinase activity, and (iii) *Bacillus cereus/Bacillus thuringiensis* agar (BCBT; R&F Products, Downers Grove, IL) to detect PI-PLC activity (70). Culturing on agar plates was performed as per the manufacturer's instructions; BHI and BCBT agar plates were incubated at 35°C for 24 h, and Bacara agar plates were incubated at 30°C for 24 h. Isolates were classified as PI-PLC positive if colonies appeared blue or PI-PLC-negative if colonies failed to produce blue pigment after 24 h at 35°C. Hemolysis was assayed as described by the Food and Drug Administration *Bacterial Analytical Manual* (35). Briefly, overnight cultures of isolates grown in BHI broth were inoculated onto Trypticase soy agar plates supplemented with 5% (vol/vol) sheep's blood (Becton Dickinson, Franklin Lakes, NY), followed by incubation at 35°C for 24 h. Inoculated plates were examined for zones of clearing, indicative of hemolysis, and were categorized as hemolytic (defined here as a visible zone of clearing) or nonhemolytic (no observable zone of clearing).

### Growth at 6°C.

To screen isolates for their ability to grow at 6°C, isolates were propagated in BHI broth for 18 to 20 h at 32°C, followed by spread plating 100 μl of an overnight culture onto BHI agar and subsequent incubation at 6°C. After 21 days, plates were examined for observable growth. The results were obtained from 2 independent experiments.

### PCR detection of toxin-encoding genes.

Bacterial DNA used as the template for PCR detection of virulence genes was extracted using Qiagen DNeasy blood and tissue kits (Qiagen, Hilden, Germany). For PCRs, approximately 5 ng of DNA template was added to a master mix containing ultrapure water, 2× GoTaq Green master mix (Promega, Madison, WI), and 0.4 μM primers (see Table 1 for primer sequences). PCRs included an initial denaturation time of 3 min at 94°C, followed by 30 cycles of amplification; each cycle consisted of denaturation at 94°C for 30 s, annealing (see Table 1 for annealing temperatures) for 30 s, elongation for 1 min at 72°C, and a final extension at 72°C for 7 min. PCR products were electrophoresed in 1% agarose gels, followed by ethidium bromide staining to check for amplification. For isolates that did not yield a PCR amplicon for a given gene, the PCR was repeated to confirm the negative PCR result. To confirm that the lack of amplification was not due to poor DNA template quality, PCR amplification of the single-copy housekeeping gene *rpoB* was performed.

**TABLE 1:** Primers used in this study

Primer name^*a*^	Primer sequence (5′ to 3′)	Annealing temp (°C)
FHblC	CCTATCAATACTCTCGCAA	45
RHblC	TTTCCTTTGTTATACGCTGC	45
FHblD	GAAACAGGGTCTCATATTCT	45
RHblD2	CTGCATCTTTATGAATATCA	45
FHblA	GCAAAATCTATGAATGCCTA	45
RHblA	GCATCTGTTCGTAATGTTTT	45
F2NheA	TAAGGAGGGGCAAACAGAAG	52
RNheA	TGAATGCGAAGAGCTGCTTC	52
F2NheB	CAAGCTCCAGTTCATGCGG	52
RNheB	GATCCCATTGTGTACCATTG	52
FNheC	ACATCCTTTTGCAGCAGAAC	52
R2NheC	CCACCAGCAATGACCATATC	52
FCytK	CGACGTCACAAGTTGTAACA	52
R2CytK	CGTGTGTAAATACCCCAGTT	52
FEntFM	GTTCGTTCAGGTGCTGGTAC	56
REntFM	AGCTGGGCCTGTACGTACTT	56

a The primers used in this study were reported previously (53).

### Whole-genome sequencing and sequence processing.

Whole-genome sequence (WGS) data were compiled from previous projects (8, 16) (our unpublished data). Sequence reads and genome assemblies were deposited in NCBI and assigned the accession numbers listed in Table S1. Virulence genes and MLST loci were extracted from processed WGS data using BTyper (44). Maximum likelihood phylogenetic trees were constructed based on alignments of *hblC* and *nheB* genes using general time-reversible (GTR) substitution model with 1,000 bootstrap iterations in RaxML version 8 (Fig. 3) (71). Core genome SNPs were detected in assembled genomes with kSNP 3.0 (72) and were used for the construction of a phylogenetic tree, as described above for single genes.

### Detection of HblC and NheB.

The Duopath cereus enterotoxins kits (Merck, Kenilworth, NJ) were used to detect HblC (the lytic component L2, a subunit of toxin Hbl) and NheB (the binding component of the Nhe toxin). Isolates were first substreaked from frozen glycerol stocks onto BHI agar, followed by incubation at 32°C for 22 h. After incubation, single colonies were inoculated into BHI broth, which was incubated at 37°C for 18 to 20 h under static conditions. For testing, cultures were adjusted to room temperature (20°C) using a water bath, and a 150-μl aliquot was then transferred to the sample port of the Duopath kit. The results were read after 30 min of incubation at room temperature. The detection limits for HblC and NheB are 20 and 6 ng/ml, respectively (73).

### Cytotoxicity.

Fifty-two isolates (i.e., all isolates except B. anthracis Ames Ancestor) were tested for cytotoxicity using a modified version of the propidium iodide (PI) exclusion assay to assess the compromised membrane integrity of HeLa cells following coincubation with supernatants of B. cereus isolates (74). This assay was selected, as Hbl, Nhe, and CytK are pore-forming toxins and hence are expected to affect the membrane integrity of HeLa cells (37, 42, 43). Bacterial culture supernatants were prepared by inoculating a single colony of each isolate into 5-ml aliquots of BHI and subsequently incubating them for 16 to 18 h at 37°C. Just prior to tissue culture cytotoxicity assays, 1 ml of overnight culture of each isolate was pelleted in a 1.5-ml Eppendorf tube by centrifugation at 6,800 × *g* for 5 min. The supernatants were transferred to clean 1.5-ml tubes and used for the tissue culture assays.

HeLa cells (0.8 × 10^5^ to 1 × 10^5^ cells/well) were seeded into 24-well plates containing 12-mm glass coverslips at 20 to 24 h prior to exposure to bacterial culture supernatants. Prior to the assays, Eagle's minimum essential medium (EMEM) in the 24-well plate was replaced with 950 μl of prewarmed EMEM per well, and plates were returned to the 37°C incubator for 1 h. Bacterial supernatants were then added (50 μl, 5% [vol/vol]) to HeLa cells, followed by incubation at 37°C with 5% CO_2_ for 15 min. The supernatant was used at 5%, as for some isolates, concentrations above 5% resulted in the detachment of all HeLa cells from the glass coverslips, a phenomenon which has been reported previously in response to other pore-forming toxins (75). Next, 5 μg of a 1 mg/ml PI stock solution (Biotium, Hayward, CA) was added to the treated HeLa cells, and cells were incubated at 37°C for an additional 10 min. The medium was subsequently removed, and cells were fixed with 4% (vol/vol) formaldehyde in PBS at room temperature for 15 min. Cells were then permeabilized with 0.5% Triton X-100 (J. T. Baker, Center Valley, PA) at room temperature for 10 min. HeLa cell nuclei were stained with 1 μg/ml 4′,6-diamidino-2-phenylindole (DAPI) solution (Thermo Fisher Scientific, Waltham, MA) at room temperature for 1 to 5 min and were subsequently rinsed with sterile deionized water. Glass coverslips were then mounted onto microscope slides using Dako Glycergel (Dako North America, Inc., Carpinteria, CA). Slides were imaged using a Zeiss 710 confocal microscope (Cornell University Biotechnology Resource Center, Bioimaging Facility), and images were processed using the FIJI software (76). The proportion of cells with decreased membrane integrity (nuclei stained with PI) relative to all cells (nuclei stained with DAPI) was averaged from 2 fields of view for each microscope slide. The data represent the results from 2 independent experiments. For statistical analyses, cytotoxicity was coded as a categorical response, with bacterial strains that showed an average of ≥5% PI-positive cells considered cytotoxic. A 5% cutoff was chosen arbitrarily to delineate cytotoxic from noncytotoxic strains.

### Statistical analyses.

All statistical analyses were performed using R statistical software version 3.2.5. Significant relationships (*P* < 0.05) between specific phenotypic and molecular characteristics and the clades were determined using Fisher's exact tests (77). Comparisons were performed to assess associations between clades and the genetic and phenotypic characterizations (e.g., proportion of isolates in clade I containing *hblCDA* versus proportion of isolates in all other clades containing *hblCDA*). Subsequent hypothesis-driven statistical testing was used to test for associations between pore formation and detection of the diarrheal toxins Hbl (subunit L2), Nhe (subunit NheB), and *cytK* (PCR detection of the *cytK-2* gene) (42). *P* values were adjusted for multiple comparisons using a false-discovery rate (FDR) correction method. All *P* values are listed in Table S2.

### Accession number(s).

Newly determined sequence accession numbers were deposited in the NCBI SRA database under accession numbers SRR3458443, SRR4064641, SRR4064645, SRR4661781 to SRR4661784, SRR4661789, SRR4661790, SRR5185009 to SRR5185011, SRR5185016, SRR5185018 to SRR5185020, SRR5185025, SRR5185026, SRR5185033, SRR5185034, SRR5185037, SRR5189056 to SRR5189059, SRR5189062, SRR5189064, and SRR5189065.

## Supplementary Material

Supplemental material
