# Supplementary material for: Intraclade Variability in Toxin Production and Cytotoxicity of Bacillus cereus Group Type Strains and Dairy-Associated Isolates
Source: Appl Environ Microbiol. 2018 Mar 1;84(6):e02479-17. doi: 10.1128/AEM.02479-17 (PMC5835744; doi:10.1128/AEM.02479-17)
Supplement: Supplemental material [file supp_84_6_e02479-17__index.html]

Supplemental material 

# Intraclade Variability in Toxin Production and Cytotoxicity of Bacillus cereus Group Type Strains and Dairy-Associated Isolates

## Supplemental material

- Supplemental file 1 -

  Phenotypic and molecular characterization of all *B. cereus* group isolates included in this study (Table S1); *P* values of statistical analyses of associations between virulence factors and phylogenetic clades and cytotoxicity (Table S2); number and proportion of PCR-positive results for toxin genes (Table S3); number of isolates with positive results for toxin genes using WGS data, within each clade (Table S4).

  PDF, 296K
