# Supplementary material for: Intraclade Variability in Toxin Production and Cytotoxicity of Bacillus cereus Group Type Strains and Dairy-Associated Isolates
Source: Appl Environ Microbiol. 2018 Mar 1;84(6):e02479-17. doi: 10.1128/AEM.02479-17 (PMC5835744; doi:10.1128/AEM.02479-17)
Supplement: Supplemental material [file AEM.02479-17_zam006188379s1.pdf]

Table S1. Phenotypic and molecular characterization of all *B. cereus* group isolates included in this study.

Isolate ID	Clade[1]	*rpoB* AT[2]	MLST Type	SR/RefSeq Accession	Growth at 6°C[3]	Hemolytic[3]	PI-PLC[3,4]	Lecithinase[3]	NheB[3,5]	Hbl-L2[3,5]	*hblA*[3]	*hblC*[3]	*hblD*[3]	*nheA*[3]	*nheB*[3]	*nheC*[3]	*cytK*[3]	*entFM*[3]	Cytotox. Average[6]
FSL H8-0534	I	148	83	SRR4661789	-	+	-	+	-	-	+	+	+	-	-	+	-	-	0.98
FSL K6-0042	I	128	87	SRR5185033	-	+	-	+	-	-	+	+	+	-	-	+	-	-	1.00
FSL H8-0481	I	128	1346	SRR4661784	-	+	-	+	-	-	+	+	+	-	-	+	-	-	0.65
FSL K6-1142	I	128	1346	SRR4661782	-	+	-	+	-	-	+	+	+	-	-	+	-	-	0.95
FSL K6-0220	I	148	83	SRR4064641	-	+	-	+	-	-	+	+	+	-	-	+	-	-	0.90
DSM 12442[T]	I	148	83	NZ_CM000745.1	-	-	-	+	-	-	+	-	+	-	-	+	-	+	0.98
FSL H7-0344	II	59	1097	SRR5185037	-	+	+	+	+	+	-	-	-	+	+	+	-	+	0.04
FSL J3-0113	II	417	1094	SRR3458443	+	+	+	+	+	-	-	+	+	+	+	+	-	+	0.00
FSL K6-0069	II	194	1080	SRR2541606	-	+	+	+	-	-	-	-	-	+	+	+	+	+	0.13
FSL M8-0091	II	410	564	SRR5189063	+	+	+	+	+	-	+	+	+	+	+	+	-	+	0.00
FSL W8-0169[T]	II	61	1081	SRR2541651	+	+	+	+	+	+	+	+	+	+	+	+	+	+	0.88
FSL K6-0067	III	365	1086	SRR2541605	-	-	+	+	+	-	-	-	-	+	+	+	+	+	0.00
FSL M8-0117	III	308	1085	SRR2541639	-	+	+	+	+	-	+	+	+	+	+	+	+	+	0.00
FSL R5-0594	III	246	1096	SRR5185018	-	+	+	+	+	+	+	+	+	+	+	+	+	+	0.01
FSL W7-1328	III	327	1093	SRR5185010	-	-	-	+	-	-	-	-	-	+	+	+	+	+	0.00
FSL W8-0003	III	125	1084	SRR2541640	-	-	+	+	+	-	-	-	-	+	+	+	-	+	0.06
FSL W8-0050	III	380	32	SRR2541641	-	+	+	+	+	-	-	-	-	+	+	+	+	+	0.06
FSL W8-0275	III	463	1050	SRR2541668	-	+	+	+	+	-	-	-	-	+	+	+	+	+	0.00
FSL W8-0483	III	120	1082	SRR2541674	-	+	+	+	+	-	-	-	-	+	+	+	-	+	0.00
FSL W8-0520	III	481	1032	SRR2541680	-	+	+	+	+	-	-	-	-	+	+	+	+	+	0.01
FSL K6-0040	IV	362	1100	SRR2541603	-	+	+	+	+	+	+	+	+	+	+	+	+	+	0.79
FSL K6-0043	IV	363	1087	SRR2541604	-	+	+	+	+	+	+	+	+	+	+	+	+	+	0.80
FSL K6-0073	IV	366	33	SRR2541607	-	+	+	+	+	+	+	+	+	+	+	+	+	+	0.04

Isolate ID	Clade[1]	*rpoB* AT[2]	MLST Type	SR/RefSeq Accession	Growth at 6°C[3]	Hemolytic[3]	PI-PLC[3,4]	Lecithinase[3]	NheB[3,5]	Hbl-L2[3,5]	*hblA*[3]	*hblC*[3]	*hblD*[3]	*nheA*[3]	*nheB*[3]	*nheC*[3]	*cytK*[3]	*entFM*[3]	Cytotoxicity Average[6]
FSL K6-0268	IV	409	230	SRR5185034	-	+	+	+	+	-	+	+	+	+	+	+	+	+	0.01
FSL K6-1030	IV	556	1191	SRR4661783	-	-	-	+	+	-	+	-	+	+	+	+	+	+	0.00
FSL M8-0139	IV	424	138	SRR5185026	-	-	+	+	+	-	-	-	-	+	+	+	+	+	0.00
FSL R5-0184	IV	277	927	SRR5185020	-	+	+	+	+	+	+	+	+	+	+	+	+	+	1.00
FSL R5-0585	IV	278	1143	SRR5185019	-	+	+	+	+	-	+	+	+	+	+	+	+	+	0.01
FSL R5-0859	IV	158	1099	SRR5185016	-	+	+	+	+	+	+	+	+	+	+	+	+	+	0.00
FSL W7-1101	IV	296	1100	SRR5185011	-	+	+	+	+	+	+	+	+	+	+	+	+	+	0.30
FSL W7-1334	IV	304	265	SRR5185009	-	+	+	+	-	+	+	+	+	+	+	+	-	+	0.00
FSL W8-0640	IV	154	1089	SRR2541688	-	+	+	+	+	+	+	+	+	+	+	+	+	+	0.00
FSL W8-0824	IV	92	24	SRR2541693	-	+	+	+	+	+	+	+	+	+	+	+	+	+	0.64
ATCC 14579^{T}	IV	158	4	NC_004722.1	-	+	+	+	+	+	+	+	+	+	+	+	+	+	0.99
ATCC 10792^{T}	IV	689	10	NC_005957.1	-	+	+	+	+	+	+	+	+	+	+	+	+	+	0.76
FSL M8-0214	V	412	223	SRR5185025	-	+	+	+	+	+	+	+	+	+	+	+	-	+	0.42
FSL H8-0482	V	129	223	SRR2541601	-	+	+	+	+	-	+	+	+	+	+	+	-	+	0.01
BCT-7112^{T}	V	129	111	NC_022781.1	-	+	+	+	+	+	+	+	+	+	+	+	-	+	0.02
FSL H7-0683	VI	75	222	SRR4064645	+	-	+	+	-	-	+	+	+	+	+	+	-	+	0.00
FSL E2-0214	VI	531	410	SRR4661781	+	+	+	+	-	-	+	+	+	-	+	+	-	+	0.00
FSL H7-0926	VI	90	667	SRR2541537	+	+	+	+	-	-	+	+	+	+	+	+	-	+	0.00
FSL H8-0485	VI	132	1142	SRR5189059	+	-	+	+	+	-	-	+	+	+	+	+	-	+	0.00
FSL H8-0492	VI	134	1098	SRR4661790	+	-	+	+	-	-	+	+	+	+	+	+	-	+	0.00
FSL J3-0123	VI	513	1095	SRR5189058	+	+	+	+	+	-	+	+	+	+	+	+	-	+	0.00
FSL M7-0109	VI	273	410	SRR5189057	+	+	+	+	+	-	+	+	+	-	+	+	-	+	0.00
FSL M7-0669	VI	3	655	SRR5189065	+	+	+	+	+	-	+	+	+	-	+	+	-	+	0.00

Isolate ID	Clade[1]	*rpoB* AT[2]	MLST Type	SR/RefSeq Accession	Growth at 6°C[3]	Hemolytic[3]	PI-PLC[3,4]	Lecithinase[3]	NheB[3,5]	Hbl-L2[3,5]	*hblA*[3]	*hblC*[3]	*hblD*[3]	*nheA*[3]	*nheB*[3]	*nheC*[3]	*cytK*[3]	*entFM*[3]	Cytotoxicity Average[6]
FSL M7-1219	VI	97	1092	SRR5189064	+	+	+	+	+	-	+	+	+	+	+	+	-	+	0.00
FSL R5-0708	VI	257	1144	SRR5189062	+	+	+	+	+	-	+	+	+	+	+	+	-	+	0.00
FSL W7-1108	VI	342	673	SRR5189056	+	+	+	+	+	-	+	+	+	+	+	+	-	+	0.00
DSM 2048[T]	VI	3	116	NZ_CP009692.1	+	+	+	+	+	-	+	+	+	-	+	+	-	+	0.00
WSBC 10204[T]	VI	3	196	NZ_CP009746.1	+	-	+	+	+	-	+	+	+	+	+	+	-	+	0.00
NVH 391-98[T]	VII	688	930	NC_009674.1	-	+	-	+	-	-	-	-	-	-	-	-	+	+	0.25

[1] Phylogenetic group determined based on concatenated 7-gene MLST sequences.
[2] AT refers to *rpoB* allelic type; determined based on 632 nucleotide sequence of *rpoB*.
[3] "+" denotes the bacterial isolate was positive for a given virulence factor, "-" denotes the virulence factor was not detected. Data presented are based on PCR amplification of target genes.
[4] PI-PLC refers to phosphoinositide phospholipase C activity as determined by plating isolates on *Bacillus cereus*/*Bacillus thuringiensis* chromogenic plating medium.
[5] Lecithinase activity determined by plating isolate on Bacara agar.
[6] Proportion of HeLa cells that were positive for propidium iodide staining, following a 30 minute co-incubation with supernatants (5% v/v) from bacterial cultures grown at 37°C. Results are averaged from two independent experiments.

Table S2. P-values of statistical analyses of associations between virulence factors and phylogenetic clades and cytotoxicity

Association tested[1,2]	P-value for overall associations[3]	P-values for significant associations with specific phylogenetic clades[4,5,6]
**Association between phylogenetic clades and virulence factors**		
PI-PLC Activity	P < 0.001	I: P < 0.001
Hemolysis	P = 0.720	ND
Growth at 6°C	P < 0.001	III: P = 0.043<br>IV: P = 0.002<br>VI: P < 0.001
hblCDA gene presence	P < 0.001	III: P < 0.001<br>VI: P = 0.047
nheABC gene presence	P = 0.032	VII: P = 0.019
entFM gene presence	P < 0.001	I: P < 0.001
cytK gene presence	P < 0.001	I: P = 0.025<br>IV: P < 0.001<br>VI: P < 0.001
Hbl-L2 Immunoassay Detection	P < 0.001	IV: P < 0.001<br>VI: P = 0.005
NheB Immunoassay Detection	P < 0.001	I: P < 0.001<br>IV: P = 0.043
Cytotoxicity	P < 0.001	I: P = 0.001<br>VI: P = 0.002
**Cytotoxicity versus presence/absence of selected genes**		
hblCDA	P = 1.000	NA
cytK	P = 0.316	NA
nheABC	P = 0.422	NA
**Cytotoxicity versus immunoassay detection of Hbl-L2 and NheB**		
Hbl-L2	P = 0.098	NA
NheB	P = 0.140	NA

[1] Includes both *cytK-1* and *cytK-2*.
[2] Considered *hblCDA* or *nheABC* positive if at least one gene in the toxin gene operon was detected via PCR.
[3] P-values from Fisher's exact tests, corrected using False Discovery Rate for multiple comparisons.
[4] P-values from Fisher's exact tests, uncorrected.
[5] ND = not determined (i.e. analysis not performed).
[6] NA = not applicable.

Table S3. Number and proportion of PCR-positive results for toxin genes.

Clade	Gene[1]															
	hblA		*hblC*		*hblD*		*nheA*		*nheB*		*nheC*		*cytK*		*entFM*	
I	6	(1.00)	5	(0.83)	6	(1.00)	0	(0.00)	0	(0.00)	6	(1.00)	0	(0.00)	1	(0.17)
II	2	(0.40)	3	(0.60)	3	(0.60)	5	(1.00)	5	(1.00)	5	(1.00)	2	(0.40)	5	(1.00)
III	2	(0.22)	2	(0.22)	2	(0.22)	9	(1.00)	9	(1.00)	9	(1.00)	7	(0.78)	9	(1.00)
IV	14	(0.93)	13	(0.87)	14	(0.93)	15	(1.00)	15	(1.00)	15	(1.00)	14	(0.93)	15	(1.00)
V	3	(1.00)	3	(1.00)	3	(1.00)	3	(1.00)	3	(1.00)	3	(1.00)	0	(0.00)	3	(1.00)
VI	12	(0.92)	13	(1.00)	13	(1.00)	9	(0.69)	13	(1.00)	13	(1.00)	0	(0.00)	13	(1.00)
VII	0	(0.00)	0	(0.00)	0	(0.00)	0	(0.00)	0	(0.00)	0	(0.00)	1	(1.00)	1	(1.00)
Total	39	(0.75)	39	(0.75)	41	(0.79)	41	(0.79)	45	(0.87)	51	(0.98)	24	(0.46)	47	(0.90)

[1] Number and proportion (in parentheses) of isolates in a given clade that were PCR-positive for gene

Table S4. Number of isolates with positive results for toxin genes using WGS data, within each clade.

Clade	Gene[1]															
	hblA		*hblC*		*hblD*		*nheA*		*nheB*		*nheC*		*cytK*		*entFM*	
I	6	(1.00)	5	(0.83)	4	(0.67)	0	(0.00)	3	(0.50)	4	(0.67)	0	(0.00)	0	(0.00)
II	2	(0.40)	3	(0.60)	3	(0.60)	5	(1.00)	5	(1.00)	5	(1.00)	2	(0.40)	5	(1.00)
III	2	(0.22)	2	(0.22)	2	(0.22)	9	(1.00)	9	(1.00)	9	(1.00)	7	(0.78)	9	(1.00)
IV	14	(0.93)	14	(0.93)	13	(0.87)	15	(1.00)	15	(1.00)	15	(1.00)	14	(0.93)	14	(0.93)
V	3	(1.00)	3	(1.00)	3	(1.00)	3	(1.00)	3	(1.00)	3	(1.00)	0	(0.00)	3	(1.00)
VI	13	(1.00)	10	(0.77)	13	(1.00)	13	(1.00)	13	(1.00)	13	(1.00)	0	(0.00)	13	(1.00)
VII	0	(0.00)	0	(0.00)	0	(0.00)	0	(0.00)	0	(0.00)	0	(0.00)	1	(1.00)	1	(1.00)
Total	40	(0.77)	37	(0.71)	38	(0.73)	45	(0.87)	48	(0.92)	49	(0.94)	24	(0.46)	45	(0.87)

[1] Proportion of isolates in a given clade that were PCR-positive for gene
